# Validation of the DLQI questionnaire in assessing the disease burden and principal aspects related to life quality of vitiligo patients

**DOI:** 10.3389/fpsyg.2024.1333723

**Published:** 2024-05-30

**Authors:** Laszlo Fekete, Laszlo Barna Iantovics, Gyula Laszlo Fekete

**Affiliations:** ^1^Doctoral School of Medicine and Pharmacy, George Emil Palade University of Medicine, Pharmacy, Science, and Technology of Targu Mures, Targu Mures, Romania; ^2^Department of Electrical Engineering and Information Technology, George Emil Palade University of Medicine, Pharmacy, Science, and Technology of Targu Mures, Targu Mures, Romania; ^3^Department of Dermatology, George Emil Palade University of Medicine, Pharmacy, Science, and Technology of Targu Mures, Targu Mures, Romania

**Keywords:** health-related quality of life, vitiligo, psychometric properties of a questionnaire, Dermatology Life Quality Index, symptoms and feelings, treatment, burden of vitiligo, Principal Axis Factoring

## Abstract

According to many studies, vitiligo has a negative psychological influence on the patient's life. Multiple factors contribute to the severity of the vitiligo disease burden, among which the most important are self-esteem, stress, and stigma. We aimed to measure the importance of health-related life quality in assessing disease burden in patients with vitiligo. We formulated an HA, which is the principal hypothesis, claiming a single fundamental factor that characterizes the life quality of patients with vitiligo. We also formulated 10 important research questions related to the quality of life that can be generally formulated for patients with dermatological illnesses but particularly suited for vitiligo patients. These research questions capture fundamental aspects of the health-related quality of life of vitiligo patients influenced by symptoms and feelings, daily activities, leisure, job and education, personal relationships, and treatment. These also cover specific aspects related to the quality of life, such as skin-caused sexual difficulties, difficulties in social relationships, and difficulties in performing sports, among others. The Dermatology Life Quality Index (DLQI) questionnaire measures the health-related quality of life of persons suffering from skin diseases. We applied this generic questionnaire to patients with vitiligo. Following a set of inclusion and exclusion criteria, we obtained 114 carefully selected patients who responded to all the questions. This study also validated the DLQI questionnaire on persons who suffer from vitiligo. We investigated whether DLQI has acceptable internal consistency by applying Cronbach's alpha internal consistency indicator (Cα). The obtained Cα = 0.914 indicates excellent internal consistency. We also examined whether all the questions in the questionnaire were mathematically consistent, which we finally proved. It was not necessary to remove any of the questionnaire questions. To prove our HA, a Principal Axis Factoring (PAF) was applied, verifying the assumptions regarding the Average Variance Extracted (AVE) and Convergent Validity (CV). HA proved that applying PAF on DLQI resulted in extracting a single general vitiligo latent factor of life quality, with an initial eigenvalue = 5.671, SS loadings = 5.2, and 52 % of the total cumulative variance explained. Diverse statistical analyses were applied to analyze the 10 formulated research questions. The results of the analysis of the research questions are presented and discussed in the manuscript. One of the conclusions related to the analysis of a research question was that sex had the lowest correlation with the latent life quality factor identified for vitiligo patients.

## 1 Introduction

Vitiligo is the most commonly acquired complex disorder of pigmentation, with a prevalence of ~1% in the world population (Ezzedine et al., 2015; Ye et al., 2022; Yu et al., 2022). It is characterized by well-circumscribed depigmented macules and patches that result from a progressive loss of functional melanocytes. Yang et al. (2021) studied the morphological alterations and increased S100B expression in epidermal Langerhans cells detected in the skin of patients who suffer from progressive vitiligo. Aguilera-Durán and Romo-Mancillas (2021) studied the behavior of chemokine receptor 6 (CXCR6) complex with CXCL16 soluble form chemokine by molecular dynamic simulations (Bhuptani et al., 2020). Factors that contribute to the severity of the vitiligo disease burden are multiple, among which the most important are quality of life, self-esteem, stress, and stigma. In the context of health and illness, quality of life is a multidimensional concept that addresses wellbeing and satisfaction with how health is affected, including emotional state, physical functioning, and social wellbeing (Simons et al., 2020). These aspects affect the patient's judgment of how the disease affects their life and the impact of the disease on their professional and personal goals (Divyesh, 2017; Elbuluk and Ezzedine, 2017). The impact of this disease on the patient's quality of life has been compared with serious pathologies, such as malignant diseases, hypertension, diabetes, and other diseases (Rapp et al., 1999; Tatu et al., 2022). Some autoimmune diseases associated with vitiligo can also impact the quality of life (Brǎnişteanu et al., 2017). One study (Brihan et al., 2020) investigated the implications of self-esteem in the quality of life of patients with psoriasis. Another (Stanescu et al., 2020) studied the extreme situations of assessment of suicidal behavior in dermatology. While one research (Arabkermani et al., 2022) studied the development of a minimum data set required to create a registry system for patients with vitiligo, another (Raimondi et al., 2023) presented a network analysis study of the life quality levels of Hidradenitis Suppurativa severity, and yet another (Clarke et al., 2022) studied the effect of self-compassion in adjusting skin conditions.

Various tools have been developed to measure the quality of life by focusing on different conditions and diseases. The Dermatology Life Quality Index (DLQI) questionnaire is designed for patients over 16 years of age (Finlay and Khan, 1994). It can be used to measure the impact of many dermatological diseases on quality of life (Ingordo et al., 2011). The DLQI questionnaire has been successfully used in many countries to study skin disease and the influence of the disease on quality of life. For instance, in a previous study (Ferraz et al., 2006), the Brazilian–Portuguese version of DLQI was used to study the impact of Lupus Erythematosus Cutaneous on the quality of life. One study (Bali et al., 2018) used the DLQI questionnaire to study the association between quality of life and clinical characteristics in patients with morphea. Another study (Rencz et al., 2022) used it to measure the properties of the ICECAP, a capability wellbeing instrument among dermatological patients.

The DLQI questionnaire is self-explanatory and can be simply handed to the patients without the need for detailed explanations. It has been translated worldwide and extensively applied.

The health status and health-related quality of life of patients with vitiligo in Romania and worldwide are understudied. We performed a study on health-related quality of life in a cohort of patients suffering from vitiligo, using the DLQI questionnaire to assess the disease burden. One of our aims was to perform a validation study of the DLQI questionnaire responses on persons who suffer from vitiligo to prove that it has appropriate internal consistency and that all the questions included in the questionnaire were internally consistent. It was not necessarily aimed at removing any of them. We used Exploratory Axis Factoring (EAF), a method of Exploratory Factor Analysis (EFA). We also formulated and analyzed 10 research questions related to the principal aspects of the life quality of vitiligo patients, including the following classes: symptoms and feelings, daily activities, leisure, job and education, personal relationships, and treatment. At the same time, this study can be considered a guide for the correct application of EAF and interpretation of the results; EAF involves many assumptions that researchers frequently fail to verify. A difficult aspect of EFA is establishing the number of factors to be extracted. In a previous study (Iantovics et al., 2019), we offered a comprehensive overview of the current state regarding the decision on the number of factors to be extracted. We presented a solution for establishing the number of factors that must be extracted. The proposal consisted of considering the total cumulative variances explained by the factors that must reach a certain value, which also depends on the specificity of the research. The decision rule used in this study is based on our previous work (Iantovics et al., 2019), which is also being presented as a validation method. We (Dobai et al., 2022) used the decision rule proposed earlier (Iantovics et al., 2019) and applied EFA to study cardiac implantable electronic devices of patients during the COVID-19 pandemic in Europe.

## 2 Materials and methods

### 2.1 Study design, survey, and participants

Patients diagnosed with vitiligo from outpatient offices and the outpatient department of the Dermatology Clinic from Târgu Mureş participated in the study, following the inclusion and exclusion criteria. The study period was between March 2021 and March 2022. Inclusion criteria: patients over 18 years of age, confirmed with vitiligo of any form, and who sign the informed consent form. Exclusion criteria: patients under 18 years of age, confirmed with vitiligo of any form, and those who do not sign the informed consent. Further, cases with missing responses to one or more questions were excluded. To carry out the study, the approval of the Ethics Commission of the Faculty of Medicine was obtained with no. 1255/2021, and that of the Mureş County Clinical Hospital with no. 16501/2021. Patients completed the DLQI (Cho, 2016) questionnaire. The questionnaire contained 10 questions, each with 4 or 5 answer options.

DLQI intends to assess the quality-of-life impairment of adult patients (aged ≥ 16 years) in the preceding week. It has 10 items covering the following six aspects of health-related quality of life: symptoms and feelings (questions 1, 2), daily activities (Q.3, 4), leisure (Q. 5, 6), work/school (Q.7), personal relationships (Q. 8, 9), and treatment (Q. 10). Each item is answered on a 4-point scale scored as follows: “not at all” = 0, “a little” = 1, “a lot” = 2, and “very much” = 3. Not relevant response (NRR) options are available for items 3–10. Item scores are added up to give a minimum score of 0 and a maximum score of 30, where a higher DLQI total score indicates a greater degree of health-related quality-of-life impairment. The Romanian version of the paper-based DLQI was used in the surveys.

The questionnaire was paper- and pencil-based, with unlimited time. At the same time, the complete dermatological clinical examination was performed voluntarily by experienced dermatologists involved. All patients signed the informed consent form. The data collected from the questionnaires and the clinical examination were entered into Excel worksheets. The data was encoded in compliance with the General Data Protection Regulation (GDPR).

### 2.2 Formulation of the research questions

In this section we introduce the 10 research questions, RQ = {RQ_1_, RQ_2_, RQ_3_, RQ_4_, RQ_5_, RQ_6_, RQ_7_, RQ_8_, RQ_9_, RQ_10_}, that were studied. Some questions are generally applicable to people with skin diseases. Based on this fact, we found them formulated in a certain way in the DLQI questionnaire. The motivations for their appropriateness for vitiligo patients are discussed. Finally, we analyze these research questions for vitiligo patients based on the survey questionnaire responses that we collected.

#### 2.2.1 For vitiligo patients, the itchy, sore, painful, or stinging skin influences the quality of life

Itchy, sore, painful, or stinging skin symptoms are present in almost all patients with vitiligo and can negatively influence their quality of life in their daily lives. Yang et al. (2022) highlighted this issue in their work.

Based on our bibliographic and observational study, the following research questions were formulated.

***RQ***_**1**_**.** ***For vitiligo patients, itchy, sore, painful, and stinging of the skin negatively influences their*** ***quality of life***.

For responding to RQ_1_, we will analyze the result of Q1 from the DLQI questionnaire.

***Q1***. *Over the last week, how itchy, sore, painful, or stinging has your skin been?*

#### 2.2.2 Vitiligo enhances embarrassment and self-consciousness

Patients with vitiligo constantly experience embarrassment and self-consciousness, especially among younger age groups. This could decrease with the chronicity of the disease, as concluded in an article by Dev et al. (2022).

***RQ***_**2**_. ***Vitiligo increases embarrassment or self-consciousness***

For responding to RQ_2_, we will analyze the result of Q2 from the DLQI questionnaire.

***Q2***. *Over the last week, how embarrassed or self-conscious have you been because of your skin?*

#### 2.2.3 Vitiligo impacts shopping or regular activities at home or in the garden

Shopping or undertaking regular activities at home can be challenged by this disease due to its visibility. It could seriously impact the person's quality of life, as Elbuluk and Ezzedine (2017) determined in their study.

***RQ***_**3**_. ***Vitiligo has a negative influence on going shopping or doing usual activities at home or in*** ***the garden***.

For responding to RQ_3_, we will analyze the result of Q3 from the DLQI questionnaire.

***Q3***. *Over the last week, how much has your skin interfered with you going shopping or looking after your home or garden?*

#### 2.2.4 Vitiligo influences the choice of clothes the patients wear

Camouflaging the disease condition by wearing appropriate clothes is important for these patients. It can increase the quality of their life worldwide, especially in some parts of the world, such as Al-Shammari et al. (2021) mentioned in a paper.

***RQ***_**4**_. *Vitiligo influences the choice of clothes that a person wears*

For responding to *RQ*_4_, we will analyze the result of Q4 from the DLQI questionnaire.

***Q4***. *Over the last week, how much has your skin influenced the clothes you wear?*

#### 2.2.5 Vitiligo impacts social and leisure activities

According to Picardo et al. (2022), social and recreational activities may be disturbed and have a negative impact due to disease-related stigma in young patients with a recently diagnosed condition.

***RQ***_**5**_**.** *Vitiligo negatively influences social or leisure activities*

For responding to *RQ*_5_, we will study the result of Q5 from the DLQI questionnaire.

***Q5***. *Over the last week, how much has your skin affected any social or leisure activities?*

#### 2.2.6 Vitiligo's influence in playing sports

Engaging in sports activities may be limited in persons with vitiligo wearing camouflage clothes because it is embarrassing for them. In time, these patients will refrain from sports activities altogether, diminishing their quality of life, as suggested by Bhandarkar and Kundu (2012).

***RQ***_**6**_**.** *Vitiligo negatively influences playing sports*

For responding to *RQ*_6_, we will analyze the result of Q6 from the DLQI questionnaire.

***Q6***. *Over the last week, how much has your skin made it difficult for you to do any sport?*

#### 2.2.7 Vitiligo impacts working or studying

In general, working or study activities do not influence the quality of life, except for those with the visible form of the disease, as concluded by Peralta-Pedrero et al. (2022).

***RQ***_**7**_ *. Vitiligo has a negative influence on working or studying*

For responding to *RQ*_7_, we will analyze the result of Q7 from the DLQI questionnaire.

***Q7***. *Has your skin prevented you from working or studying over the last week? If “No,” how much has your skin been a problem at work or studying?*

#### 2.2.8 Vitiligo influences social relationships with partners, close friends, or relatives

At the beginning stages of the disease, a severe negative influence on the quality of life can appear in social relations, which in time will decrease, especially with partners and close friends, as highlighted by Alikhan et al. (2011).

Based on the bibliographic study and the observational study that we performed, the following research question can be formulated.

***RQ***_**8**_**.** *Vitiligo creates social problems with the partner, friends, or relatives*.

For responding to *RQ*_8_, we will study the result of Q8 from the DLQI questionnaire.

***Q8***. *Over the last week, how much has your skin created problems with your partner or any of your close friends or relatives?*

#### 2.2.9 Vitiligo influences sex

Usually, sexual activities are not influenced by the disease in the case of married people. However, for those who would like to establish a new relationship, this issue could be a problem, as described by Bibeau et al. (2022).

***RQ***_**9**_**.** *Vitiligo can cause sexual difficulties*

For responding to *RQ*_9_, we will study the result of Q9 from the DLQI questionnaire.

***Q9***. *Over the last week, how much has your skin caused sexual difficulties?*

#### 2.2.10 Vitiligo treatment can make homes messy or take up time

On patients with chronic dermatoses, such as vitiligo, local treatment difficulties do not have a severe impact on quality of life, as concluded by Cortés et al. (2022).

***RQ***_**10**_**.** *Vitiligo has a negative influence on making the home messy or taking up time*

For responding to *RQ*_10_, we will study the result of Q10 from the DLQI questionnaire.

***Q10***. *Over the last week, how much of a problem has the treatment for your skin been, for example, by making your home messy or by taking up time?*

### 2.3 Statistical analyses

Table 1 presents the interpretation of the internal consistency that we calculated using the Cronbach alpha (Cα) (Cho, 2016) reliability indicator. For additional validation, we used the McDonald's Omega coefficient value (ω) = 0.915 (McDonald, 1999).

**Table 1:** Internal consistency interpretation.

**Cα**	**Internal consistency classification**
Cα ≥ 0.9	Excellent
0.8 ≤ Cα < 0.9	Good
0.7 ≤ Cα < 0.8	Acceptable
0.6 ≤ Cα < 0.7	Questionable
0.5 ≤ Cα < 0.6	Poor
Cα < 0.5	Unacceptable

Initially, a statistical scale Cα reliability analysis was performed on the questionnaire outcomes. The data normality assumption of the variables was realized using the Lilliefors (Lill) test, which is an adaptation of the Kolmogorov-Smirnov goodness-of-fit test (Dallal and Wilkinson, 1986). For visual validation of the normality analysis result, the interpretation of the Q-Q plot (Wilk and Gnanadesikan, 1968) visual representation was performed. This was applied for each of the variables (these representations are not included in this paper). For the verification of each variable's influence on the internal consistency, we calculated the value of Cα in case each variable was removed. The correlations between each of the variables were analyzed. We applied nonparametric correlation coefficient r calculus (Stigler, 1989; Bonett and Wright, 2000). For each case/patient, the measured quality of life using the DLQI questionnaire with results retained in the variable ResultLifeQuality was calculated. On the ResultLifeQuality variable, we performed descriptive statistics and normality analysis, finally calculating the correlation of all the variables that constituted the DLQI.

Table 2 presents the usual interpretation of the Kaiser–Meyer–Olkin measure of sampling adequacy (KMO) test results that are adopted in many types of research (Hauben et al., 2017).

**Table 2:** Interpretation of Kaiser–Meyer–Olkin (KMO) measure results.

**KMO value**	**Classification of the sampling adequacy**
0.9 ≤ KMO ≤ 1	Marvelous
0.8 ≤ KMO < 0.9	Meritorious
0.7 ≤ KMO < 0.8	Middling
0.6 ≤ KMO < 0.7	Mediocre
0.5 ≤ KMO < 0.6	Miserable
0 ≤ KMO < 0.5	Unacceptable

Based on the principle that a causal model exists, we performed a Principal Axis Factoring (PAF) (Prather et al., 1997; Grieder and Steiner, 2022) that can be used to determine the underlying latent factor structure of a set of variables. We analyzed that for the interpretation of the results, it is necessary to have a certain rotation in case more factors can be extracted. We verified all the necessary assumptions for the correct application of the PAF, including the value of the determinant of a correlation matrix, which should be higher than 0.0001 (Field, 2000), Kaiser–Meyer–Olkin Measure (KMO) (Hauben et al., 2017) test assumption, where KMO ≥ 0.6, Bartlett's Test of Sphericity (BTS) (Hauben et al., 2017) test assumption, corresponding to the *p*-value of BTS, where *p* < 0.05.

Additionally, we analyzed the anti-image matrices to see if there were candidate variables for removal (Hauben et al., 2017). We applied the decision rule that each value from the diagonal must pass the threshold by 0.6; otherwise, the corresponding variable was to be removed.

For an in-depth study, we analyzed the communalities (McDonald, 1985). Initial communalities represent the proportion of variance accounted for in each variable by the rest of the variables. Extracted communalities are estimations of the variance in each variable accounted for by the factors in the factor solution. It must be noted that the extracted communality is specific to each variable; it represents the common variance explained in each variable by the extracted factors. As a decision rule for removing a variable, we considered the extracted communality that has a value lower than or equal to 0.2.

For the precise establishment of the number of factors, we considered the decision rule based on the following combination of criteria (Choi et al., 2017; Iantovics et al., 2019):

(A) the Kaiser rule that claims the selection of only those factors that have eigenvalues ≥ 1 (Kaiser, 1960);

(B) visual interpretation of the Scree plot (Cattell, 1966) by searching for the inflation points; and

(C) considering the total cumulative variances explained by the factors we indicated in previous research (Iantovics et al., 2019).

This rule was applied and then validated using Parallel Analysis, a simulation based on the Monte Carlo method (Horn, 1965).

Finally, the Corresponding Residuals (Hauben et al., 2017) and the Reproduced Correlations (Hauben et al., 2017) were calculated. There were verified assumptions regarding the Average Variance Extracted (AVE) (Nia et al., 2020) and Convergent Validity (CDV) (Nia et al., 2020).

## 3 Results

In this section, we share the results obtained and discuss them.

### 3.1 Participant characteristics

Richard et al. (2022) presented a population-based study of the prevalence of the most common skin diseases in Europe. Vitiligo is a rare condition found in 0.5%−4% of the world's population (Krüger and Schallreuter, 2012; Al-Harbi, 2013). The highest incidence has been reported in India, followed by Mexico and Japan (Sehgal and Srivastava, 2007).

The 114 patients in our study were all Caucasians (people from the Caucasus) with phototypes between I-III on the Fitzpatrick's skin type scale. Of these, 56 (49.12%, 95% CI 39.81%−58.44%) were male, and 58 (50.88%, 95% CI 41.56%−60.19 %) were female. The mean age was 49, with 95% CI [45.8, 52.3], a median of 49, between 19 and 78 years, and a range of 59. Most of the patients, 76 (66.67%), lived in urban areas, and 38 (33.33%) in rural areas. Regarding marital status, 77 (67.54%) patients were married, and 37 (32.46%) were unmarried.

### 3.2 Descriptive statistics and data reliability analysis

Experimental setup: number of items/questions/variables = 10, denoted LQ = {LQ_1_, LQ_2_, LQ_3_, LQ_4_, LQ_5_, LQ_6_, LQ_7_, LQ_8_, LQ_9_, LQ_10_}; number of valid cases n = 114, 100%, no missing values in the questions. Cα = 0.915 indicates an excellent internal consistency, 0.915 > 0.9. Table 3 shows the results of the descriptive statistics of the LQ variables. Each variable takes a value between 0 and 4, with the significance of increasing effect with each unit increase. The rest of the columns are labeled as follows: Mean presents the mean; SD denotes the standard deviation; 95% CI of mean represents the 95% CI of the mean; Min denotes the smallest; Max denotes the largest; Range is calculated as max-min; Median denotes the median. mean_LQ_ denotes the average of LQ, with mean_LQ_ = 1.331

**Table 3:** Descriptive statistical characterization of the LQ variables.

**Variable**	**Mean**	**SD**	**95% CI of mean**	**Min**	**Max**	**Range**	**Median**
LQ_1_	1.25^<^	1.201	1.02–1.47	0	3	3	1
LQ_2_	1.62^>^	1.229	1.39–1.85	0	3	3	1.5
LQ_3_	1.23^<^	1.22	1–1.45	0	3	3	1
LQ_4_	1.45^>^	1.284	1.21–1.69	0	3	3	2
LQ_5_	1.55^>^	1.249	1.32–1.78	0	3	3	1.5
LQ_6_	1.05^<^	1.268	0.82–1.29	0	3	3	0
LQ_7_	1.37^~^	1.397	1.11–1.63	0	3	3	1
LQ_8_	1.35^~^	1.255	1.12–1.58	0	3	3	1
LQ_9_	1.45^>^	1.377	1.19–1.7	0	3	3	1
LQ_10_	0.99^<^	0.99	0.77–1.22	0	3	3	0

^>^Denotes a variable mean higher than mean_*LQ*_; ^<^denotes a variable mean lower than mean_*LQ*_; ^~^denotes a variable mean equal to mean_*LQ*_; SD, Standard Deviation; CI, confidence interval.

As a next step, we studied the statistical differences between the means of the variables. First, we verified the LQ data normality using the Shapiro-Wilk test (Shapiro and Wilk, 1965) because the LQ sample size is small. The obtained p (*p*-value), *p* = 0.767 (*p* > 0.05), indicates that the normality assumption passed. Based on this fact, it is appropriate to apply the Grubbs (Stefansky, 1972) outlier detection (the studied LQ variable passed the expected normality). The Grubbs test has the specificity that each application can detect a single statistical outlier or a value that is not an outlier but is statistically furthest from the rest. At the first application, it detected (0.99), representing the mean of LQ_10_ as a value that is furthest from the rest. In the applications that followed, the Grubbs test, by removing the previously identified furthest from the rest, detected only other values that were furthest from the rest. Removing LQ_10_ and applying it again detected 1.05 (mean of LQ_6_). Removing LQ_6_ detected LQ_2_. Removing LQ_2_ detected LQ_5_. Removing LQ_5_ detected LQ_3_. Removing LQ_3_ detected LQ_1_. Removing LQ_1_ detected LQ_8_. Removing LQ_8_ detected LQ_7_. For the remaining LQ_4_ and LQ_9_, it was not possible to apply the Grubbs test since its application requires at least three values.

For the LQ_1_, LQ_2_,…..LQ_10_ variables normality analysis was undertaken using the Lilliefors test (Table 4). According to the results, none of the variables LQ_1_, LQ_2_,…..LQ_10_ passed the assumption of normality even at the 0.001 significance level (*p* < 0.001). For final visual validation, we used the Q-Q plot representation for each studied variable (the manuscript does not present Q-Q plot representations). The visual analysis of the normality of each variable led to the same conclusion as the numerical evaluation results.

**Table 4:** Lilliefors test results.

**Variable**	**Statistic**	**Degrees of freedom**	***p*-value**
LQ_1_	0.245	114	0
LQ_2_	0.246	114	0
LQ_3_	0.255	114	0
LQ_4_	0.247	114	0
LQ_5_	0.228	114	0
LQ_6_	0.341	114	0
LQ_7_	0.301	114	0
LQ_8_	0.236	114	0
LQ_9_	0.283	114	0
LQ_10_	0.336	114	0

To verify each variable's influence on the scale consistency, the value of Cronbach's alpha for each variable was calculated (Table 5), and the column labeled “Cronbach's Alpha if Item Removed.” The differences of these values from Cα = 0.915 show the amount of change in the reliability in case of variable deletion.

**Table 5:** Cronbach's alpha if an item/variable is removed.

**Variable**	**Cronbach's alpha if item removed**	**Cα - Cronbach's alpha if item removed**
LQ_1_	0.910	0.004
LQ_2_	0.903	0.011
LQ_3_	0.903	0.011
LQ_4_	0.902	0.012
LQ_5_	0.905	0.009
LQ_6_	0.906	0.008
LQ_7_	0.903	0.011
LQ_8_	0.905	0.009
LQ_9_	0.909	0.005
LQ_10_	0.910	0.004

Cα = 0.914.

Table 6 presents the nonparametric Spearman correlation analysis of all the LQ variables since none passed the normality assumption. r denotes the calculated Spearman correlation coefficient. All the correlations were significant at the 0.01 significance level (2-tailed). p denotes the obtained *p*-value based on whether the correlation is significant.

**Table 6:** Non-parametric correlations between LQ variables.

		**LQ_1_**	**LQ_2_**	**LQ_3_**	**LQ_4_**	**LQ_5_**	**LQ_6_**	**LQ_7_**	**LQ_8_**	**LQ_9_**	**LQ_10_**
LQ_1_	*r*	1	0.609	0.473	0.490	0.405	0.427	0.457	0.615	0.307	0.439
	*p*	-	0	0	0	0	0	0	0	0	0
LQ_2_	*r*	0.609	1	0.598	0.545	0.604	0.448	0.661	0.628	0.4	0.448
	*p*	0	-	0	0	0	0	0	0	0	0
LQ_3_	*r*	0.473	0.598	1	0.659	0.592	0.545	0.624	0.472	0.563	0.527
	*p*	0	0	-	0	0	0	0	0	0	0
LQ_4_	*r*	0.49	0.545	0.659	1	0.582	0.618	0.589	0.485	0.572	0.472
	*p*	0	0	0	-	0	0	0	0	0	0
LQ_5_	*r*	0.405	0.604	0.592	0.582	1	0.521	0.594	0.501	0.490	0.462
	*p*	0	0	0	0	-	0	0	0	0	0
LQ_6_	*r*	0.427	0.448	0.545	0.618	0.521	1	0.638	0.506	0.561	0.42
	*p*	0	0	0	0	0	-	0	0	0	0
LQ_7_	*r*	0.457	0.661	0.624	0.589	0.594	0.638	1	0.581	0.47	0.462
	*p*	0	0	0	0	0	0	-	0	0	0
LQ_8_	*r*	0.615	0.628	0.472	0.485	0.501	0.506	0.581	1	0.476	0.493
	*p*	0	0	0	0	0	0	0	-	0	0
LQ_9_	*r*	0.307	0.4	0.563	0.572	0.49	0.561	0.47	0.476	1	0.463
	*p*	0.001	0	0	0	0	0	0	0	-	0
LQ_10_	*r*	0.439	0.448	0.527	0.472	0.462	0.42	0.462	0.493	0.463	1
	*p*	0.001	0	0	0	0	0	0	0	0	-

r denotes the correlation coefficient; p denotes the p-value.

LQI is the variable that centralizes the DLQI questionnaire test results. Table 7 presents the descriptive statistical characterization of LQI.

**Table 7:** Descriptive statistical characterization of LQI.

**Indicator**	**Statistic**	**Standard error**
Mean	2.46	0.14
95% CI for the mean	2.18- 2.73	
5% Trimmed mean	2.51	
Median	3.00	
Variance	2.233	
SD	1.494	
Lowest	0	
Highest	4	
Range	4	
Interquartile range	3	
Skewness	−0.546	0.226
Kurtosis	−1.148	0.449

**Figure 1:**
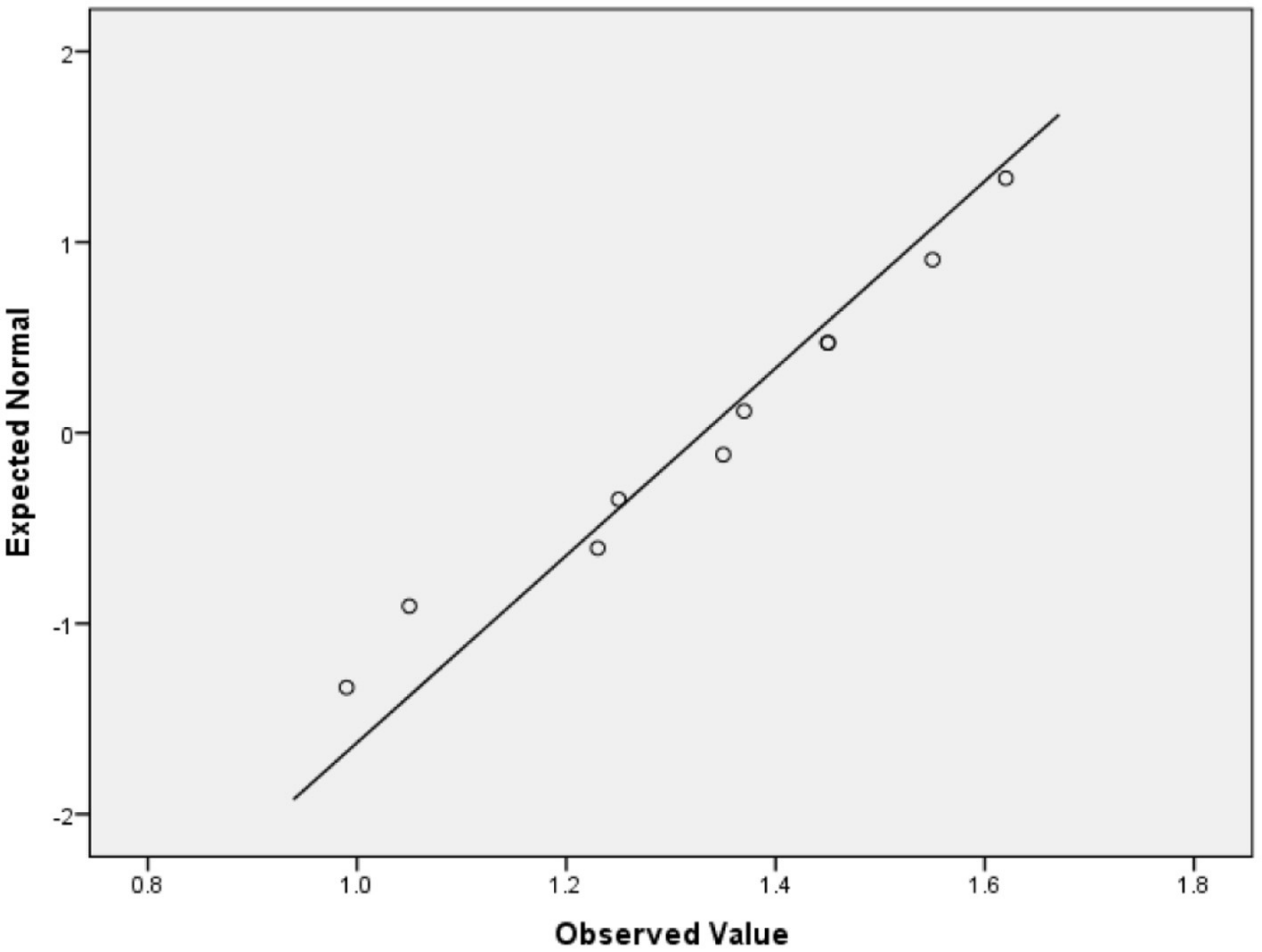
Q-Q plot of LQI for visual validation of the normality.

To verify if the LQI variable was normally distributed, we applied the Lilliefors (Lill) (Lilliefors, 1969) (Table 8) and the Shapiro-Wilk (SW) tests (Razali and Wah, 2011) (Table 8) since the SW test power is highest than those of most other normality tests (Hu and Bentler, 1999). The Lill and SW test results indicated that LQI failed to pass the normality assumption even at the 0.001 significance level. Additionally, the Q-Q plot of LQI (Figure 1) visually validated the numerical normality test results.

**Table 8:** Lilliefors and Shapiro–Wilk normality test results for the LQI variable.

	**Lilliefors**			**Shapiro-Wilk**		
	**Statistic**	**Degrees of freedom**	***p*** **-value**	**Statistic**	**Degrees of freedom**	***p*** **-value**
Score	0.230	114	0	0.831	114	0

Table 9 shows the nonparametric correlation of the variable LQI with each studied variable LQ_1_, LQ_2_……LQ_10_. We calculated the Spearman r correlation coefficient. To verify the statistical significance of r, we calculated the 95% CI. Each correlation was proved statistically significant even at the 0.001 significance level (2-tailed).

**Table 9:** Spearman correlations between LQ_1_, LQ_2_,……LQ_10_ and LQI.

**Results**	**LQ_1_**	**LQ_2_**	**LQ_3_**	**LQ_4_**	**LQ_5_**	**LQ_6_**	**LQ_7_**	**LQ_8_**	**LQ_9_**	**LQ_10_**
r	0.674	0.748	0.782	0.769	0.755	0.735	0.796	0.73	0.692	0.665
*p-*value	0	0	0	0	0	0	0	0	0	0
95% CI of r	0.556–0.766	0.651–0.821	0.696–0.846	0.679–0.837	0.66–0.826	0.634–0.812	0.714–0.856	0.628–0.808	0.579–0.78	0.545–0.759

r denotes the correlation coefficient; CI, confidence interval.

**Figure 2:**
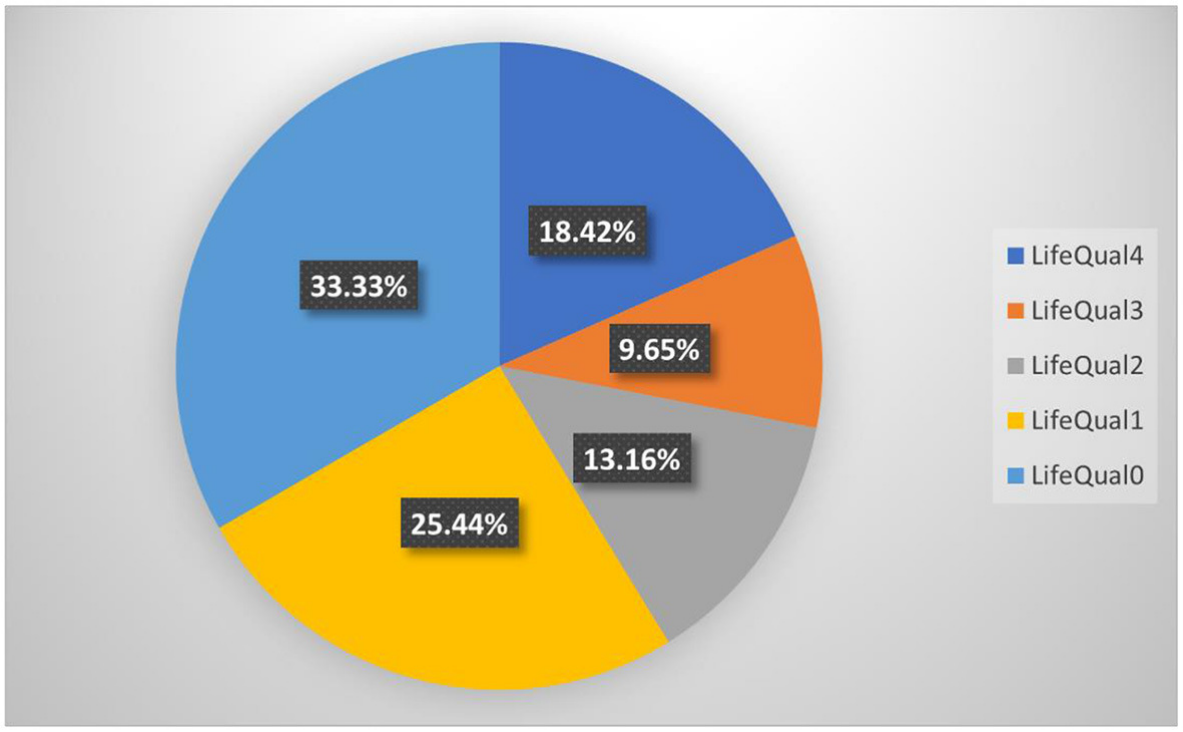
Vitiligo influence on life quality percentual distribution.

After that, we created a dummy variable denoted LQIClass with the following values: LifeQual0, indicating without effect on the life quality; LifeQual1, indicating a low effect on the life quality; LifeQual2, indicating a moderate effect on the life quality; LifeQual3, indicating an important effect on the life quality; and LifeQual4, indicating a very important effect on the life quality. As seen in Figure 2, there were 38 respondents (33.33%) with LifeQual0, 29 (25.44%) with LifeQual1, 15 (13.16%) with LifeQual2, 11 (9.65%) with LifeQual3, and 21 (18.42%) with LifeQual4.

## 4 Principal Axis Factoring for identifying the latent factor

Based on the principle that a causal model exists, we performed Principal Axis Factoring (PAF) without any rotation premised on the initial hypothesis that a single factor exists. Preliminarily, all the mandatory assumptions necessary for the correct application of PAF were applied. To avoid premature convergence, we set the maximum iterations for convergence to 1000, the usual value being 25.

### 4.1 Verification of the assumption that the PAF application is correct

The determinant of the correlation matrix of the variables LQ_1_,….LQ_10_ was 0.004, which passed the assumption to be higher than 0.0001. KMO = 0.912 test assumption passed, KMO = 0.912 >0.6, corresponding to Table 2, can be considered marvelous. The BTS test passed, and *p* = 0 (approximate chi-square = 611.298, df = 45) indicated significance. It must be mentioned that this test indicates significance in the case of a large sample size. Here, it indicated that our data is accurate based on the relatively smaller sample size.

The values from the diagonal of the anti-image matrices (Table 10) passed the threshold by 0.5 (even 0.6), which shows that none of them should be removed.

**Table 10:** The anti-image correlation matrices.

**Variable**	**LQ_1_**	**LQ_2_**	**LQ_3_**	**LQ_4_**	**LQ_5_**	**LQ_6_**	**LQ_7_**	**LQ_8_**	**LQ_9_**	**LQ_10_**
**LQ**_**1**_	0.870	−0.303	−0.089	−0.136	0.039	−0.101	0.129	−0.358	0.157	−0.072
**LQ**_**2**_	−0.303	0.89	−0.189	−0.053	−0.237	0.146	−0.289	−0.212	0.05	0.04
**LQ**_**3**_	−0.089	−0.189	0.92	−0.232	−0.108	−0.028	−0.181	0.167	−0.207	−0.166
**LQ**_**4**_	−0.136	−0.053	−0.232	0.938	−0.123	−0.221	−0.079	0.038	−0.193	−0.042
**LQ**_**5**_	0.039	−0.237	−0.108	−0.123	0.954	−0.081	−0.107	−0.033	−0.098	−0.081
**LQ**_**6**_	−0.101	0.146	−0.028	−0.221	−0.081	0.908	−0.306	−0.08	−0.229	0.032
**LQ**_**7**_	−0.129	−0.289	−0.181	−0.079	−0.107	−0.306	0.91	−0.162	0.06	−0.037
**LQ**_**8**_	−0.358	−0.212	0.167	0.038	−0.033	−0.08	−0.162	0.888	−0.193	−0.199
**LQ**_**9**_	0.157	0.05	−0.207	−0.193	−0.098	−0.229	0.06	−0.193	0.902	−0.159
**LQ**_**10**_	−0.072	0.04	−0.166	−0.042	−0.081	0.032	−0.037	−0.199	−0.159	0.945

The filled color is the indication of the matrix diagonal.

Table 11 presents the initial and extracted communalities. All the items passed the threshold by 0.2 (communality after extraction). Based on this result, removing any of them is not necessary.

**Table 11:** Initial and extracted communalities.

**Variable**	**Initial**	**Extraction**
LQ_1_	0.513	0.412
LQ_2_	0.635	0.589
LQ_3_	0.593	0.598
LQ_4_	0.584	0.606
LQ_5_	0.506	0.533
LQ_6_	0.529	0.498
LQ_7_	0.603	0.606
LQ_8_	0.573	0.519
LQ_9_	0.499	0.449
LQ_10_	0.39	0.392

### 4.2 Establishment of the number of factors that must be extracted

For the identification of the number of factors, we considered the decision rule composed of the following criteria:

(A) the Kaiser rule should be selected just for the factors that have eigenvalues ≥ 1;

(B) the Scree plot visual interpretation by searching for the inflation point;

(C) the total cumulative variance is explained according to the indications (Iantovics et al., 2019).

**Figure 3:**
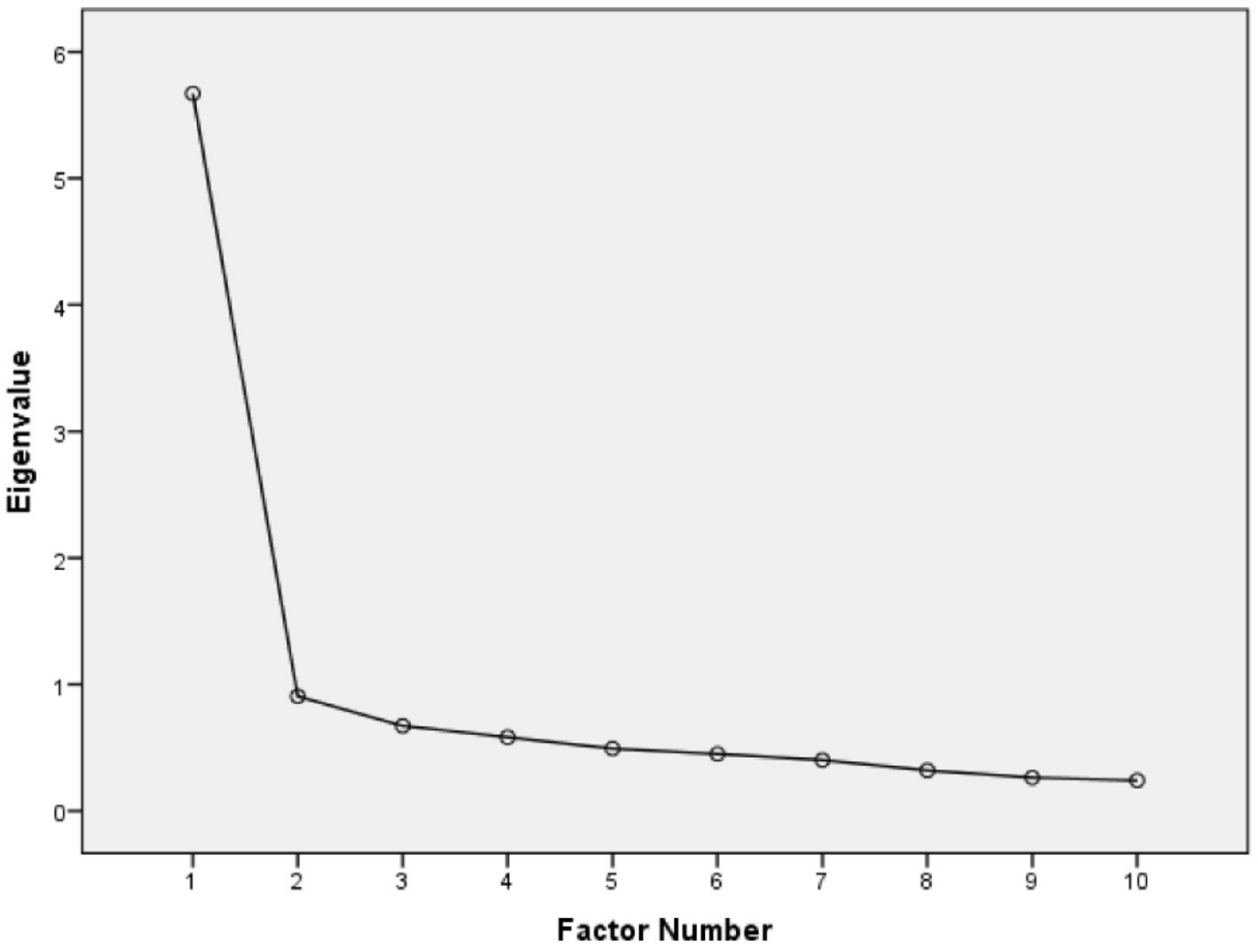
Scree plot used for visual evaluation of the number of factors.

Table 12 shows the results of factor extraction, which includes, among other things, the eigenvalues of the extracted prospective factors and the percentage of cumulative variance. Figure 3 presents the Scree plot. Applying the decision rules extracted a single factor, denoted VitiligoLifeQuality (VLQ), with an initial eigenvalue = 5.671, SS Loadings = 5.2, and 52% total cumulative variance explained.

**Table 12:** Factor extraction and variance explained.

**Factor**	**Initial eigenvalues**			**Extraction sums of squared loadings**		
	**Total**	**% of variance**	**Cumulative %**	**Total**	**% of variance**	**Cumulative %**
VitiligoLifeQuality1	5.671	56.707	56.707	5.201	52.012	52.012
2	0.906	9.063	65.770			
3	0.672	6.718	72.489			
4	0.582	5.825	78.314			
5	0.493	4.926	83.240			
6	0.45	4.500	87.740			
7	0.401	4.015	91.755			
8	0.321	3.205	94.960			
9	0.263	2.633	97.593			
10	0.241	2.407	100.000			

The cells with color background indicate the identified factor and the related Extraction sums of squared loadings.

Since only one factor could be extracted, we consider VLQ to be the only factor that can characterize the level of quality of life. We named the latent factor VLQ general life quality factor.

### 4.3 Validation of the number of factors

**Figure 4:**
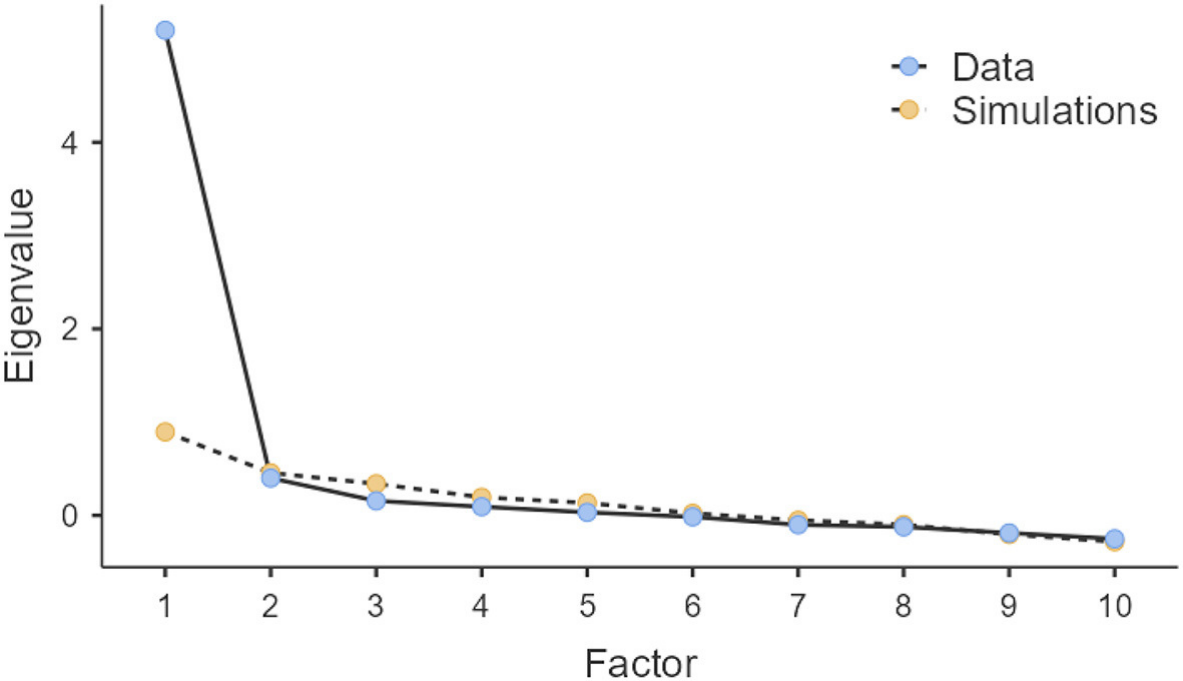
Scree plot for visual interpretation of parallel analysis results.

A single factor was obtained to validate the correctness of the number of extracted factors. We performed a parallel analysis based on Monte Carlo simulation (Table 13) with the following parameters: 900 replications, 10 variables, and 114 subjects. The columns labeled Eigenvalue number, Random Eigenvalue, and Standard Deviation present the results of the Monte Carlo simulation. The column labeled “Prospective factor number indicates the prospective factor number, which, if the decision passes, is indicated to be selected. The column labeled Initial eigenvalues represents the initial eigenvalues that are shown in Table 13. The last column, labeled Decision rule, presents the result of the decision rule applied for selecting a factor with a certain number. ^*^Indicates a value that is lower than 0.5 (Figure 4 also includes the representation of these values). According to the decision rule, if the eigenvalue after extraction is greater than the random eigenvalue, then the corresponding factor to the eigenvalue number should be selected. However, the number of selected factors using the decision rule was validated. Figure 4 shows the Scree plot for visual interpretation of the parallel analysis results.

**Table 13:** Results of parallel analysis using Monte Carlo simulation.

**Prospective factor number**	**Random eigenvalue**	**SD**	**Eigenvalue after extraction**	**Result of decision rule**
1	1.4967	0.0841	5.671	**Passed**
2	1.3299	0.0607	^*^	Not passed
3	1.2082	0.0497	^*^	Not passed
4	1.1107	0.0374	^*^	Not passed
5	1.0157	0.036	^*^	Not passed
6	0.9358	0.0331	^*^	Not passed
7	0.8548	0.037	^*^	Not passed
8	0.7741	0.0392	^*^	Not passed
9	0.6836	0.04	^*^	Not passed
10	0.5905	0.0448	^*^	Not passed

SD denotes the standard deviation. The cells with color background indicate the factor that passed the decision rule.

Since a single factor was extracted, it was not necessary to apply a rotation that could be oblique or orthogonal; however, the decision not to make rotation proved to be correct.

### 4.4 Obtaining the factor loadings

Table 14, the Factor Matrix, presents the factor loadings and the proportion of variance accounted for by the factor. In Table 14, the variables are ordered descending based on the value of loading into the factor. According to Table 11, LQ_10_ has the lowest extracted communality with a value of 0.392, but this value is appropriate to others and is much higher than 0.2. According to Table 14, all the factor loadings passed the threshold of 0.5, including LQ_10_, which was 0.626, 0.626 > 0.5. More precisely, for a sample size of 100, the required factor loading should be at least 0.55 (for a sample size of 120, the factor loading should be 0.5). All the factor loading passed this threshold. Based on this result, it can be concluded that it was not necessary to remove any of the variables. Since a single factor was identified, using the usual visual Factor Plot in the Rotated Factor Space representation was not necessary.

**Table 14:** Factor matrix.

**Variable**	**VLQ**
LQ_7_	0.778
LQ_4_	0.778
LQ_3_	0.773
LQ_2_	0.768
LQ_5_	0.73
LQ_8_	0.721
LQ_6_	0.705
LQ_9_	0.670
LQ_1_	0.642
LQ_10_	0.626

### 4.5 Validity and model fit

We calculated the Reproduced Correlations (Table 15) and the corresponding residuals (Table 16). ^*^Represents the reproduced communalities. Table 16 shows the values of residuals that are computed between observed and reproduced correlations.

**Table 15:** Reproduced correlations.

**Variable**	**LQ_1_**	**LQ_2_**	**LQ_3_**	**LQ_4_**	**LQ_5_**	**LQ_6_**	**LQ_7_**	**LQ_8_**	**LQ_9_**	**LQ_10_**
**LQ**_**1**_	0.412^*^	0.493	0.496	0.499	0.469	0.453	0.5	0.462	0.430	0.402
**LQ**_**2**_	0.493	0.589^*^	0.594	0.597	0.561	0.542	0.598	0.553	0.514	0.481
**LQ**_**3**_	0.496	0.594	0.598^*^	0.602	0.565	0.545	0.602	0.557	0.518	0.484
**LQ**_**4**_	0.499	0.597	0.602	0.606^*^	0.568	0.549	0.606	0.561	0.521	0.487
**LQ**_**5**_	0.469	0.561	0.565	0.568	0.533^*^	0.515	0.568	0.526	0.489	0.457
**LQ**_**6**_	0.453	0.542	0.545	0.549	0.515	0.498^*^	0.549	0.508	0.472	0.442
**LQ**_**7**_	0.5	0.598	0.602	0.606	0.568	0.549	0.606^*^	0.561	0.521	0.487
**LQ**_**8**_	0.462	0.553	0.557	0.561	0.526	0.508	0.561	0.519^*^	0.483	0.451
**LQ**_**9**_	0.43	0.514	0.518	0.521	0.489	0.472	0.521	0.483	0.449^*^	0.419
**LQ**_**10**_	0.402	0.481	0.484	0.487	0.457	0.442	0.487	0.451	0.419	0.392^*^

The cells with color background indicate the reproduced communalities.

**Table 16:** Residuals corresponding to the reproduced correlations.

**Variable**	**LQ_1_**	**LQ_2_**	**LQ_3_**	**LQ_4_**	**LQ_5_**	**LQ_6_**	**LQ_7_**	**LQ_8_**	**LQ_9_**	**LQ_10_**
**LQ**_**1**_		0.128	−0.033	−0.015	−0.047	−0.037	−0.058	0.161	−0.117	0.012
**LQ**_**2**_	0.128		0.007	−0.047	0.043	−0.103	0.051	0.067	−0.105	−0.045
**LQ**_**3**_	−0.033	0.007		0.05	0.014	−0.015	0.013	−0.113	0.048	0.025
**LQ**_**4**_	−0.015	−0.047	0.05		0.005	0.062	−0.017	−0.077	0.061	−0.02
**LQ**_**5**_	−0.047	0.043	0.014	0.005		−0.008	0.014	−0.036	0.009	−0.005
**LQ**_**6**_	−0.037	−0.103	−0.015	0.062	−0.008		0.064	−0.019	0.092	−0.039
**LQ**_**7**_	−0.058	0.051	0.013	−0.017	0.014	0.064		0.001	−0.046	−0.037
**LQ**_**8**_	0.161	0.067	−0.113	−0.077	−0.036	−0.019	0.001		−0.006	0.056
**LQ**_**9**_	−0.117	−0.105	0.048	0.061	0.009	0.092	−0.046	−0.006		0.064
**LQ**_**10**_	0.012	−0.045	0.025	−0.02	−0.005	−0.039	−0.037	0.056	0.064	

According to the results, 33.33% of the residuals are non-redundant, with absolute values greater than the threshold of 0.05. This finding indicates the passing of the threshold by 60%, which we considered 33.33% < 60%.

Using the results obtained in Table 11, we computed the AVE and performed a construct validity by making a CV validity verification. The average loading VLQ was 0.7191 (calculated as the average of values from column VLQ from Table 14), which passed the threshold by 0.7. The variance extracted from VLQ was 0.51710481 (calculated as the square of 0.7191, 0.7191^2^), which passed the threshold by 0.5. This proved the convergent validity assumption to be true.

A model fit measure analysis was performed. The root mean square error of approximation (RMSEA) and Tucker–Lewis index (TLI) were calculated. TLI = 0.917, TLI > 0.9, which indicates a good model fit for one factorial design (Fabrigar et al., 1999). RMSEA = 0.0952, RMSEA 90%CI = 0.0638 - 0.128. indicates an acceptable model fit (Kline, 1998).

A model test with Df = 35 was performed, obtaining CMIN = 71.5, *p* < 0.001. Where CMIN denotes the χ2 value. CMIN/Df = 2.04, 2.04 < 3 indicates acceptable fit (Tabachnick and Fidell, 2007) (it corresponds even to the constraint that is considered 2 in some research).

## 5 Discussions

### 5.1 Discussion on research questions regarding the validation of the questionnaire

Cα = 0.914 obtained for the DLQI questionnaire shows excellent internal scale consistency. ω = 0.915 allows the formulation of the same conclusion. The results presented in Table 5 prove that removing any of the variables does not significantly influence the modification of internal consistency. Based on this result, the questions are mathematically consistent. According to Table 14, since all the factor loadings are positive, it can be concluded that the numbering/coding of the values that can be chosen in the questions is realized appropriately (we did not use reverse coding for any of the variables).

### 5.2 Discussion on the hypothesis HA and the correct application of PFA

We presented and verified all the necessary assumptions that must be passed for the application of PFA to be correct (Iantovics et al., 2019). The selection of the optimal number of factors is a complex problem. Many researchers have studied this problem. We presented a proposed decision rule for selecting the appropriate number of factors and also presented a method for validating the number of extracted factors.

Applying PFA, a single factor was obtained that proved the hypothesis HA of the existence of a single life quality factor LQV. According to Table 11, the factor accounted for 52% of the variance, which is considered appropriate.

### 5.3 Discussion on the research questions: RQ_1_, RQ_2_, RQ_3_, RQ_4_, RQ_5_, RQ_6_, RQ_7_, RQ_8_, RQ_9_, and RQ_10_

Table 3 presents a descriptive statistical characterization of the LQ variables, which are questions formulated in the DLQI questionnaire. According to the results, the min = 0, max = 3, and range = 3 were the same for all the variables. This indicates that the responses were diverse, including all the values 0, 1, 2, and 3 in the case of all the variables. The median of each of the variables was 0 or 1. Each of the LQ variables has a specific standard deviation.

Initial communalities represent the proportion of variance accounted for in each variable by the rest of the variables. Extracted communalities are estimates of the variance in each variable accounted for by the factors in the factor solution. The extracted communality is specific to each variable; it represents the common variance explained in each variable by the factors. According to Table 11, the lowest extracted communality is for LQ_10_, 0.392; it represents the low common variance explained in the variable by the latent factor. This value is much lower than other variables.

Table 14 presents the factor loading matrix, showing the correlation between variables and the factors. LQ_7_ (Load 0.778), LQ_4_ (Load 0.778), LQ_3_ (Load 0.773), LQ_2_ (Load 0.768), LQ_5_ (Load 0.73), LQ_8_ (Load 0.721), LQ_6_ (Load 0.705), LQ_9_ (Load 0.67), LQ_1_ (Load 0.642), and LQ_10_ (Load 0.626). It is evident that the variables LQ_7_, LQ_4_, LQ_3_, LQ_2_, LQ5, LQ8, and LQ6 have the highest factor loadings, between 0.705 and 0.778. They are followed by the variables LQ_9_, LQ_1_, and LQ_10_, with factor loadings between 0.626 and 0.670. Based on the specificity of the questions and the correlation between them, we made the following classifications.

With r = 0.609, the correlation between LQ_1_, LQ_2_ is significant (p = 0). Based on this result and the specifics of the questions, we classified RQ_1_ and RQ_2_ as symptoms and feelings. With *r* = 0.659, the correlation between LQ_3_, LQ_4_ is also significant (*p* = 0). Based on this and the specifics of the questions, we classified RQ_3_ and RQ_4_ as daily activities. The correlation between RQ_5_ and RQ_6_ was *r* = 0.521, which was also significant (*p* = 0). Based on this fact and the specifics of the questions, we classified RQ_5_ and RQ_6_ as leisure.

## 6 Conclusions

Dermatological diseases, which are often externally visible, particularly vitiligo, have a significant negative impact on the quality of life of these patients. In this research, we studied the life quality of vitiligo patients using data provided by the validated questionnaire Dermatology Life Quality Index (DLQI). The Principal Axis Factoring clearly indicated a single factorial structure, a latent factor that we called the vitiligo life quality index (VLQ) that captures the entire phenomenology of life quality. The Average Variance Extracted and Convergent Validity assumptions were passed.

The psychometric properties of the DLQI questionnaire proved to have excellent internal consistency. The statistical analysis of the results provided by the DLQI questionnaire included questions that proved to be internally consistent. Based on this result, our study also validated this questionnaire on vitiligo. In conclusion, we can say that the questions are consistent and the answers valid. The performed EAF analysis was necessary and important for correctly interpreting the results.

We formulated 10 research questions that apply generally to people with skin diseases. Motivations were presented for their appropriateness for vitiligo. We analyzed and discussed the results based on the DLQI questionnaire. Finally, we analyzed these research questions for vitiligo patients based on the survey questionnaire responses that we collected.

At the same time, this study gives some extended explanations for the correct application of the EAF, which involves many assumptions that frequently are missed and need to be verified by researchers. It also involves some decisions, such as establishing the number of factors to be extracted that are often difficult (Choi et al., 2017; Iantovics et al., 2019). As a decision rule, for the establishment of the correct number of extracted factors, we presented a combination of criteria that included the Kaiser criterion, Scree plot visual interpretation, and Total Variance Explained (criterion previously approached by us Iantovics et al., 2019), followed by validation using parallel analysis using Monte Carlo simulation.

## Data availability statement

The raw data supporting the conclusions of this article will be made available by the authors, without undue reservation.

## Ethics statement

The studies involving humans were approved by the Ethics Commission of the Faculty of Medicine with no. 1255/2021, respectively of the Mureş County Clinical Hospital with no. 16501/2021. The studies were conducted in accordance with the local legislation and institutional requirements. The participants provided their written informed consent to participate in this study.

## Author contributions

LF: Conceptualization, Formal analysis, Investigation, Methodology, Resources, Validation, Visualization, Writing – original draft, Writing – review & editing. LI: Conceptualization, Data curation, Formal analysis, Methodology, Resources, Software, Supervision, Validation, Visualization, Writing – original draft, Writing – review & editing. GF: Conceptualization, Formal analysis, Funding acquisition, Investigation, Methodology, Project administration, Resources, Supervision, Validation, Visualization, Writing – original draft, Writing – review & editing.
